# The evolution of radiographers—an investigation into radiographers’ role and perspectives: a EuroAIM/EFRS survey

**DOI:** 10.1186/s13244-026-02442-x

**Published:** 2026-10-30

**Authors:** Patrizia Cornacchione, Moreno Zanardo, Christina Malamateniou, Helle Precht, Katy June Knight, Francesco Sardanelli, Karen Knapp

**Affiliations:** 1 Fondazione Policlinico Universitario Agostino Gemelli IRCCS, L.go A. Gemelli 8, 00168 Rome, Italy; 2 Università Cattolica del Sacro Cuore, Largo Francesco Vito 1, 00168 Rome, Italy; 3 Department of Life Sciences, Health and Health Professions, Link Campus University, Casale di San Pio V 44, 00165 Rome, Italy; 4 Unit of Radiology, IRCCS Galeazzi—Sant’Ambrogio Hospital, Cristina Belgioioso 173, 20157 Milan, Italy; 5 CRRAG Research Group, City St George’s University of London, London, ECV1 0HB UK; 6 European Federation of Radiographer Societies, Cumieira, 5030-058 Portugal; 7 European Society of Medical Imaging Informatics, Vienna, 1010 Austria; 8 King’s College London, London, SE59RS UK; 9 UCL University College, Niels Bohrs Allé 1, 5230 Odense M, Denmark; 10 University of Southern Denmark, Campusvej 55, 5230 Odense M, Denmark; 11 Lillebælt Hospital, Kolding, University Hospital of Southern Denmark, Sygehusvej 1, 6000 Kolding, Denmark; 12 University College Cork, T12 K8AF Cork, Ireland; 13 St Luke’s Campus, Heavitree Road, Exeter, EX1 2 LU UK; 14 Lega Italiana per la Lotta Contro i Tumori (LILT) Milano Monza Brianza, Piazzale Gorini 22, 20133 Milan, Italy

**Keywords:** Artificial intelligence, Professional role, Technology, Radiographer, Surveys and questionnaires

## Abstract

**Abstract:**

Radiographers play a central role within clinical imaging and radiotherapy teams, acting as a key interface between patients and increasingly complex technologies. As healthcare systems evolve, their scope of practice is expanding alongside advances in digital workflows, artificial intelligence (AI) and new models of care. This EuroAIM/EFRS survey provides a comprehensive European overview of radiographers’ roles, professional autonomy, research engagement and perspectives on AI. Based on responses from 1548 radiographers across multiple countries, the findings highlight substantial clinical autonomy and strong responsibility for image quality optimisation and radiation protection, alongside limited but growing involvement in research activities. More than half of the participants expressed interest in greater research participation, indicating a gap between current opportunities and professional aspirations. Responses regarding AI were generally positive, with many participants expecting it to enhance their professional roles, although expectations regarding its workforce impact remained heterogeneous. The survey also confirms the lasting effects of the COVID-19 pandemic on professional practice and highlights variability in perceived career development opportunities. These findings describe a profession in transition, characterised by increasing responsibilities and technological integration and underscore the need for continued investment in education, structured career frameworks and research capacity to support radiographers in an increasingly complex healthcare environment.

**Key Points:**

***Question*** What are radiographers’ current roles, autonomy, research engagement and perspectives on emerging technologies within a changing imaging and radiotherapy workforce?

***Findings*** In 1548 participants, radiographers reported substantial autonomy and responsibility, limited research involvement, positive but heterogeneous AI expectations and variable career opportunities.

***Critical Relevance Statement*** This EuroAIM/EFRS survey informs radiology practice by identifying radiographers’ professional development, education and research needs and by supporting structured career frameworks and interprofessional workforce planning.

**Graphical Abstract:**

**Figure:**
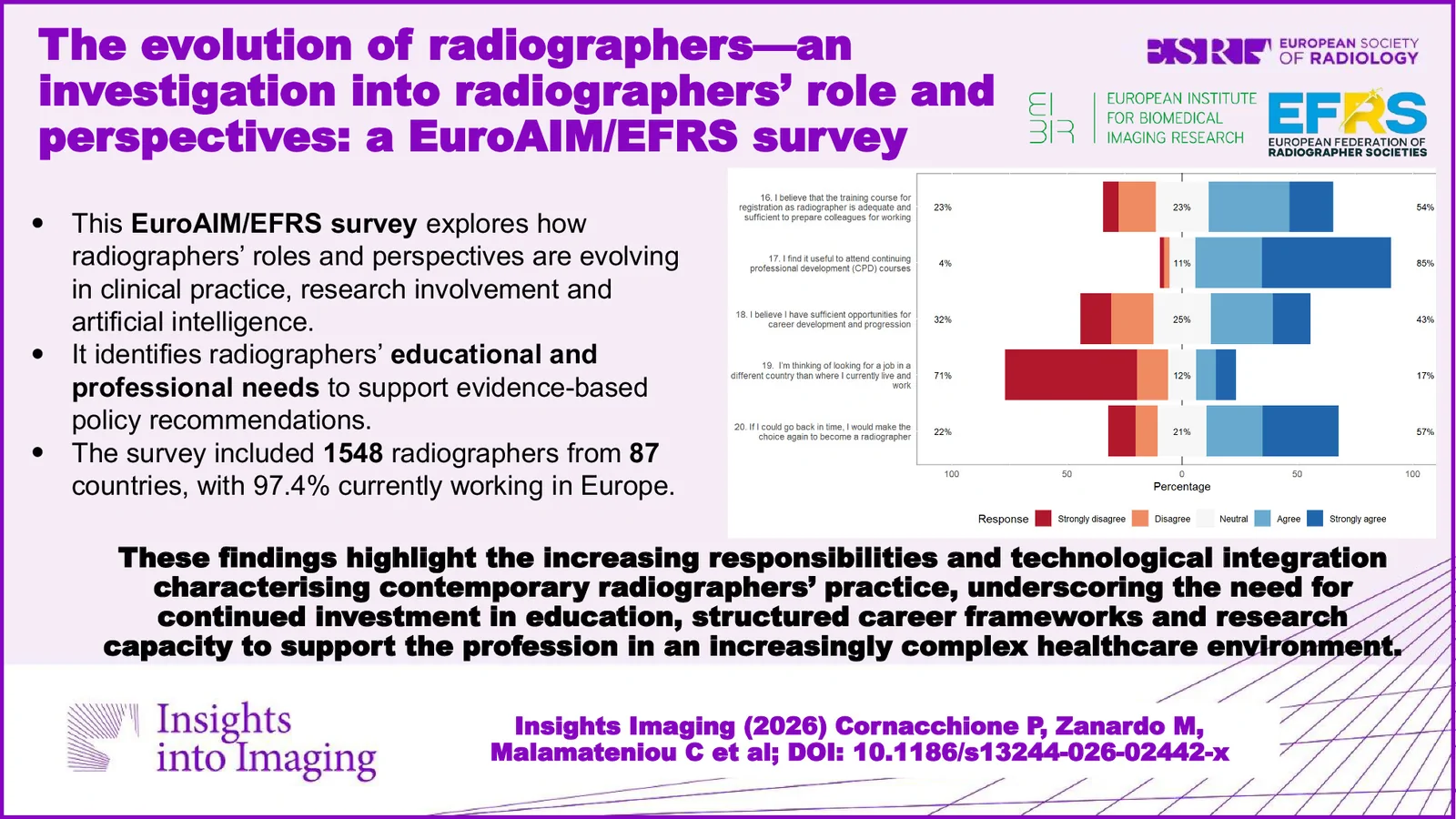

## Background

Radiographers are essential professionals in clinical imaging and radiotherapy teams, serving as an interface between patients and advanced technologies, with diagnostic radiographers involved in the care of 90% of patients [1]. In the past, their responsibilities included image acquisition, optimisation and radiation protection, but their scope of practice has expanded in parallel with technological innovations within healthcare systems, progressively including them in the clinical decision-making process [2–5]. The continuing development of imaging technologies, including advanced acquisition techniques, new imaging equipment, digital workflows and artificial intelligence (AI) tools, is reshaping radiology and radiotherapy practice and influencing radiographers’ roles, responsibilities and professional development [6, 7].

In this context, the European Federation of Radiographer Societies (EFRS) documents on role development, education, and continuous professional development [8, 9] have outlined structured career frameworks and emphasised the contribution of advanced and extended practice roles that combine expert clinical work with leadership, education, research, and service innovation [10, 11]. Empirical work across Europe confirms that these roles are increasingly implemented in both diagnostic and therapeutic radiography, but also highlights heterogeneity in titles, responsibilities, educational requirements and recognition between countries and service settings [12–14]. For instance, the United Kingdom has a robust four-tier structure with assistant practitioners, practitioners, advanced practitioners and consultant practitioners, with a remit to work across the four pillars of clinical practice, education, research and leadership within these roles [15, 16]. A fifth grouping of enhanced practitioners was later added to recognise those who are working across all four pillars, but demonstrate senior expertise in one pillar, most commonly the clinical pillar [17].

Previous EuroAIM/ESR surveys have explored the impact of AI in radiology, documenting growing familiarity with AI tools, heterogeneous usage patterns, and evolving opinions on issues such as clinical responsibility, patient acceptance and training needs among ESR members [7, 18]. However, these studies were designed primarily from the perspective of radiologists or focused on AI-related issues rather than the broader evolution of different professional roles [6, 19]. These changes are likely to influence the recognition of new skills, interprofessional collaboration, research engagement and professional identity. However, Europe-wide data about how these challenges are perceived by radiographers remains limited.

Unlike previous surveys focusing on single domains such as AI adoption or advanced practice, the current study provides an integrated overview of clinical autonomy, research engagement, workforce mobility and perceptions of emerging technologies across primarily Europe, offering a comprehensive workforce perspective. The survey was primarily conducted by the European Network for the Assessment of Imaging in Medicine (EuroAIM), a joint initiative of the European Institute for Biomedical Imaging Research (EIBIR), in collaboration with the EFRS, under the umbrella of the European Society of Radiology (ESR). In this context, EuroAIM provided the methodological and organisational framework for an evidence-generating survey relevant to imaging practice, while EFRS contributed radiography-specific professional expertise. Therefore, the present survey aimed to capture radiographers’ current roles, autonomy, research engagement and views on AI and future professional development.

## Materials and methods

### Design

Based on the anonymous design and the nature of the study (a survey of health professionals), formal ethics committee approval was deemed unnecessary in accordance with ESR data protection standards and the General Data Protection Regulation (GDPR) [20]. The study was conducted in accordance with the Declaration of Helsinki. This was an anonymous, voluntary, web-based survey using a convenience sampling approach. The survey was developed by EuroAIM in collaboration with the EFRS, under the umbrella of the ESR and EIBIR.

The working group that developed the question framework consisted of the EuroAIM Board and radiographers on the EFRS expert list who served on the EFRS Research Committee.

### Population

Eligibility criteria for participation included being a qualified radiographer and being willing to complete the survey voluntarily; no financial or non-financial incentives were offered. These criteria were stated in the introductory survey information, and participants confirmed them by clicking the “Next” button before accessing the questionnaire. Participants could opt out by not proceeding beyond the introductory information page or by closing the questionnaire before submission.

### Sampling and sample size

The survey targeted radiographers and equivalent professional titles reached through ESR and EFRS communication channels, including mailing lists, newsletters, social media and professional networks. As the questionnaire link was disseminated through multiple open channels, the total number of individuals who accessed or viewed the survey could not be determined; therefore, an exact response rate could not be calculated. No formal sample size calculation was performed, as this was an exploratory descriptive survey based on voluntary participation and convenience sampling.

### Questionnaire development, validity, and pilot testing

The questionnaire was conceptually based on previous EuroAIM/ESR surveys [7, 18], which provided a methodological framework and examples of question structure. For the present study, the survey was substantially redesigned to address the specific aims of investigating radiographers’ roles, research engagement and views on AI. During the development and review process, the survey was also submitted to relevant external reviewers within the ESR for consideration, who provided further comments, requested clarifications and suggested refinements. This was a cross-sectional survey and was not designed to measure temporal change through repeated observations. Instead, it explored radiographers’ current perceptions of professional transformation in relation to clinical autonomy, research engagement, AI, the impact of COVID-19, education and career development.

All closed questionnaire items were mandatory and had to be completed before submission; therefore, no missing data were present for the analysed survey items. The final open-ended comment box was optional and could be left blank. The full questionnaire is available as Supplementary material.

The survey consisted of three main sections comprising a total of 28 items:

1. Introduction and consent to participate. Consent was considered implicit when participants chose to proceed with the questionnaire in line with the study objectives.
2. Demographics (seven items). Items covered age range, gender, country of birth, country of education and current country of practice, highest qualification and main field(s) of work (diagnostic imaging, radiotherapy, nuclear medicine, and hybrid roles).
3. Radiographers’ role and perspectives (21 items). Items explored the scope of practice, perceived autonomy, involvement in protocol optimisation, research involvement, views on AI, and the impact of the COVID-19 pandemic. Questions were grouped into clinical activities (six items), research (three items), AI (four items), COVID-19 (two items) and education/career development (six items).

Most items were multiple-choice or multiple-response questions, with selected five-point Likert-type scales (e.g., from “completely disagree” to “completely agree”) to capture attitudes and expectations. The final open-ended comment box (item 21, Section 3) could be left blank.

The questionnaire did not undergo formal validation, and no separate pilot study was conducted.

### Recruitment and enrolment

The survey was implemented using the Google Forms platform (https://docs.google.com/forms/) and was open from July 2, 2025, to September 15, 2025, for a total duration of 11 weeks. The questionnaire was administered in English only. Participation was voluntary, and no financial or other incentives were offered. An invitation e-mail containing a brief description of the study aims and a direct survey link was distributed through ESR mailing lists, EFRS communication channels and newsletters, targeting radiographers across Europe. Two reminder e-mails were sent at predefined intervals to increase the response rate. Precisely two emails were distributed through ESR mailing lists on July 2 and August 29, 2025 to a total of 3230 radiographers who have been registered as ESR members. In parallel, on July 11, 2025, the survey link was posted on EFRS social media channels (LinkedIn, Instagram, Facebook), and a newsletter was sent to all EFRS members (51 scientific societies and professional bodies representing approximately 110,000 radiographers). A reminder on all channels and a new newsletter were sent in the last week of August 2025. Team members were invited to reshare the survey through their professional networks to support wider dissemination and maximise participation.

### Data analysis

Survey responses were automatically recorded via Google Forms and exported as comma-separated values (CSV) files. Data were subsequently analysed using Microsoft Excel (Microsoft Corp.) and RStudio Version 2025.05.1 Build 513 (R Foundation for Statistical Computing). Descriptive statistics were used to summarise demographic characteristics and survey responses. Categorical variables are reported as absolute frequencies and percentages. To maintain anonymity, we reported country-level results only when *n *≥ 5; categories with *n *< 5 were aggregated as ‘Other’. For Likert-scale items, responses were analysed as ordinal variables and presented as distributions across the five response categories (“completely disagree” to “completely agree”).

No imputation was performed for missing data, as all survey items were mandatory prior to submission. Results are presented using tables and bar charts to illustrate response distributions across thematic domains. Given the exploratory nature of the study, no inferential statistical testing was planned.

## Results

### Survey participants and demographics

A total of 1548 radiographers completed the survey and were included in the analysis. As the survey was distributed through multiple channels, including social media, an exact response rate could not be calculated. Most participants identified as female (66.2%, *n* = 1025), followed by male (32.8%, *n* = 507); a small proportion identified as non-binary or preferred not to disclose their gender (1.0%, *n* = 16 combined). Participants’ age ranged widely and was broadly distributed across age groups, with relatively similar proportions across most five-year categories between 26 and 60 years; specifically, 18–25 years (5.4%, *n* = 84), 26–30 (10.0%, *n* = 155), 31–35 (14.8%, *n* = 229), 36–40 (13.4%, *n* = 208), 41–45 (13.8%, *n* = 213), 46–50 (10.5%, *n* = 163), 51–55 (11.4%, *n* = 177), 56–60 (10.9%, *n* = 169), 61–65 (7.3%, *n* = 113), 66–70 (2.0%, *n* = 31) and ≥ 71 (0.4%, *n* = 6). The distribution was broadly balanced within the core working-age sample, with fewer participants at the youngest and oldest ages. Participants were born, educated and practising across a wide range of European countries. The UK represented the largest proportion of participants, followed by Italy, Denmark, Portugal and Albania, resulting in a geographically diverse European cohort (Table 1).

**Table 1:** Countries of birth, study, and work

Country	Birth	Study	Work
Albania	54 (3.5%)	52 (3.4%)	44 (2.8%)
Australia	8 (0.5%)	7 (0.5%)	-
Austria	21 (1.4%)	21 (1.4%)	21 (1.4%)
Belgium	-	-	5 (0.3%)
Croatia	5 (0.3%)	-	-
Denmark	86 (5.6%)	89 (5.7%)	90 (5.8%)
Estonia	13 (0.8%)	13 (0.8%)	12 (0.8%)
Finland	29 (1.9%)	31 (2.0%)	32 (2.1%)
France	7 (0.5%)	7 (0.5%)	6 (0.4%)
Germany	30 (1.9%)	28 (1.8%)	38 (2.5%)
Ghana	6 (0.4%)	-	-
Greece	20 (1.3%)	19 (1.2%)	15 (1.0%)
India	29 (1.9%)	29 (1.9%)	17 (1.1%)
Ireland	31 (2.0%)	15 (1.0%)	30 (1.9%)
Israel	-	-	5 (0.3%)
Italy	226 (14.6%)	234 (15.1%)	213 (13.8%)
Kenya	6 (0.4%)	-	-
Latvia	10 (0.6%)	8 (0.5%)	7 (0.5%)
Lithuania	31 (2.0%)	31 (2.0%)	31 (2.0%)
Malta	22 (1.4%)	23 (1.5%)	24 (1.6%)
Netherlands	20 (1.3%)	19 (1.2%)	17 (1.1%)
Nigeria	14 (0.9%)	12 (0.8%)	-
Norway	9 (0.6%)	10 (0.6%)	11 (0.7%)
Philippines	11 (0.7%)	4 (0.3%)	-
Poland	5 (0.3%)	-	-
Portugal	55 (3.6%)	55 (3.6%)	42 (2.7%)
South Africa	14 (0.9%)	15 (1.0%)	-
Spain	22 (1.4%)	25 (1.6%)	26 (1.7%)
Sweden	6 (0.4%)	6 (0.4%)	6 (0.4%)
Switzerland	40 (2.6%)	41 (2.6%)	63 (4.1%)
United Kingdom	626 (40.4%)	681 (44.0%)	747 (48.3%)
Zimbabwe	12 (0.8%)	5 (0.3%)	-
Other^a^	80 (5.2%)	60 (3.9%)	46 (3.0%)

Percentages calculated on total respondents (*n* = 1548)

^a^ Other includes any countries where the answer was < 5 for any of the questions as listed below: Algeria, Argentina, Aruba, Bangladesh, Bolivia, Bosnia and Herzegovina, Brazil, Canada, Chile, Colombia, Costa Rica, Cyprus, Czech Republic, Egypt, Georgia, Gibraltar, Guernsey, Hong Kong, Hungary, Iceland, Iraq, Isle of Man, Macedonia, Malaysia, Mauritius, Mexico, Namibia, Nepal, New Zealand, Northern Mariana Islands, Pakistan, Réunion, Romania, Russia, Saudi Arabia, Serbia, Slovenia, Somalia, Sri Lanka, Sudan, Swaziland, Syria, Uganda, Ukraine, United Arab Emirates, United States, Uruguay, Uzbekistan, Venezuela, Yemen, Zambia, all with < 5 responses

Of note, country of birth, country of education, and current country of work were collected to descriptively explore possible professional mobility among radiographers. However, the numbers were too small for most countries to draw robust conclusions regarding movement within Europe or between Europe and other regions. Although some descriptive patterns may suggest mobility towards countries such as the United Kingdom and Switzerland, these observations remain exploratory and should not be interpreted as evidence of defined migration flows. Regarding educational background, 56.7% (*n* = 877) of participants reported holding a Radiography/Medical Imaging or equivalent qualification, 26.9% (*n* = 417) of participants reported holding a Postgraduate Master’s degree in radiography, 7.8% (*n* = 121) a Master’s degree in a non-radiography field, and 4.4% (*n* = 68) a PhD qualification. All participants were qualified radiographers or equivalent professionals; the 56.7% figure refers only to those who reported Radiography/Medical Imaging or an equivalent entry-level professional qualification as their highest educational qualification. Additional responses (4.2%, *n* = 65) included other qualifications that could not be unambiguously categorised, reflecting differences in educational pathways, professional titles and qualification systems across countries.

### Fields of professional practice

Participants reported working across multiple imaging and therapeutic domains. Conventional radiography was the most frequently reported area of practice (49.1%, *n* = 760), followed by computed tomography (39.0%, *n* = 604) and magnetic resonance imaging (30.7%, *n* = 475). Other areas included interventional radiology (19.0%, *n* = 294), mammography (14.5%, *n* = 224), ultrasound (13.2%, *n* = 204), research (12.9%, *n* = 200) and radiotherapy (11.6%, *n* = 180), reflecting the diversity of contemporary radiographer practice.

### Clinical role and perceived autonomy

**Fig. 1:**
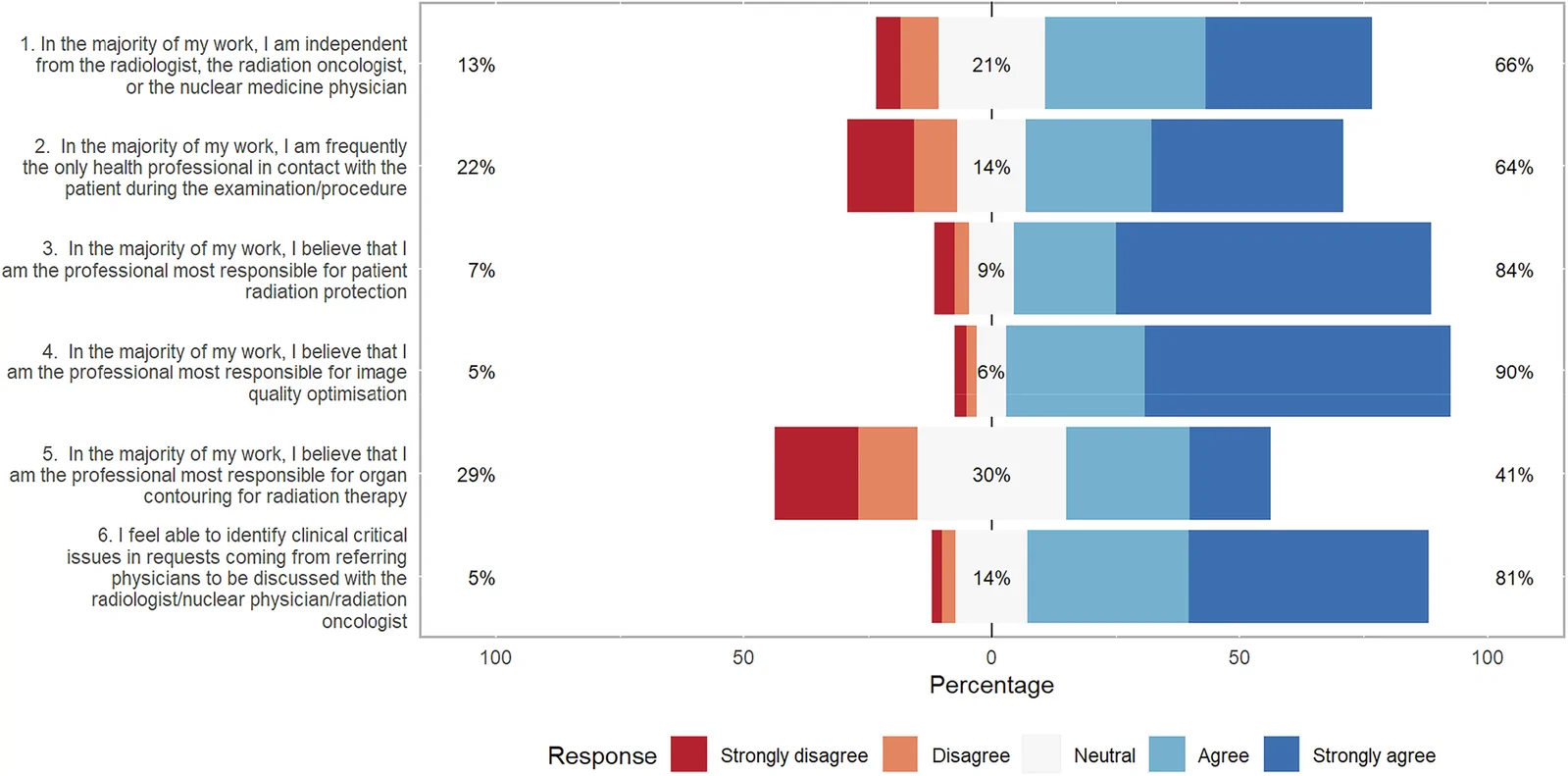
Responses to the clinical questions

Most participants reported a substantial degree of autonomy in their daily clinical practice. Overall, 66.0% (*n* = 1021) agreed or completely agreed that they operate independently from radiologists, radiation oncologists or nuclear medicine physicians, while 21.4% (*n* = 331) reported a neutral position and 12.7% (*n* = 196) disagreed. A majority of participants (64.0%, *n* = 991) agreed that they are the only healthcare professional in direct contact with the patient during imaging examinations or procedures. Responsibility for patient radiation protection was particularly high, with 84.0% (*n* = 1301) of participants agreeing or completely agreeing with this statement. Similarly, 85.8% (*n* = 1328) reported being responsible for optimising image quality, with more than half expressing complete agreement. Furthermore, 80.8% (*n* = 1251) stated that they are able to identify clinical issues in imaging requests and discuss them with the referring physician (Fig. 1).

### Research involvement

**Fig. 2:**
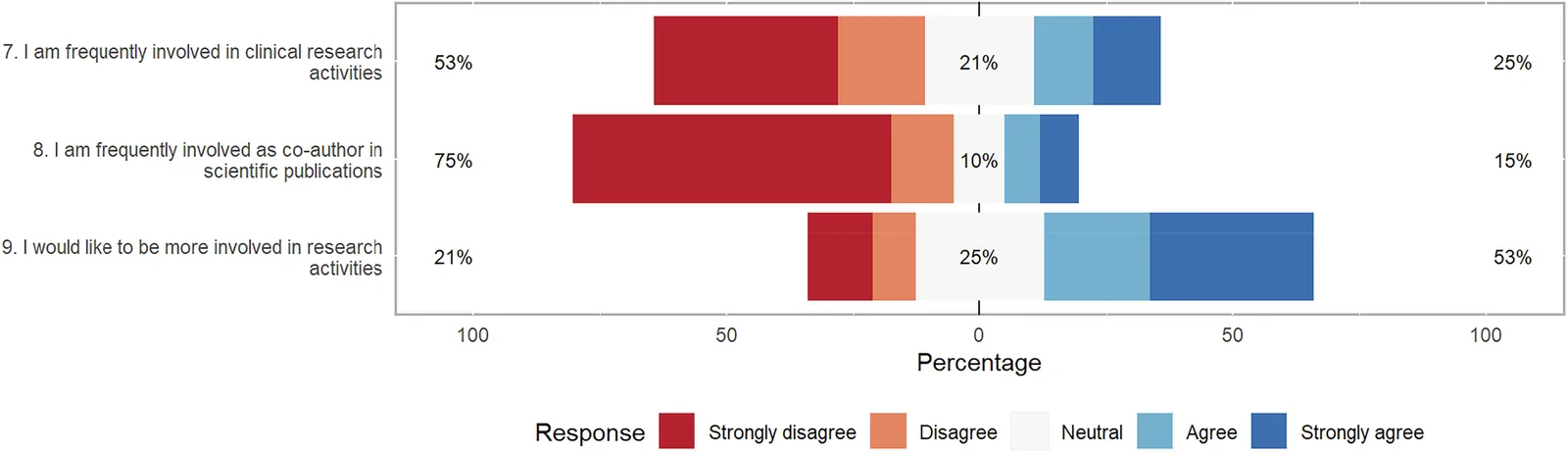
Responses to the research questions

Research engagement showed a mixed picture. While more than half of participants (53.5%, *n* = 828) disagreed or completely disagreed with the statement “I frequently participate in clinical research activities”, a substantial proportion (25.1%, *n* = 389) reported agreement, indicating that one in four radiographers is already actively involved in clinical research. Engagement in scientific publications was lower, with 75.5% (*n* = 1169) reporting no regular involvement in publishing activities. Despite this, 53.4% (*n* = 827) of participants expressed a clear interest in greater participation in research, indicating a marked discrepancy between current involvement and future aspirations (Fig. 2).

### Perceptions of artificial intelligence

**Fig. 3:**
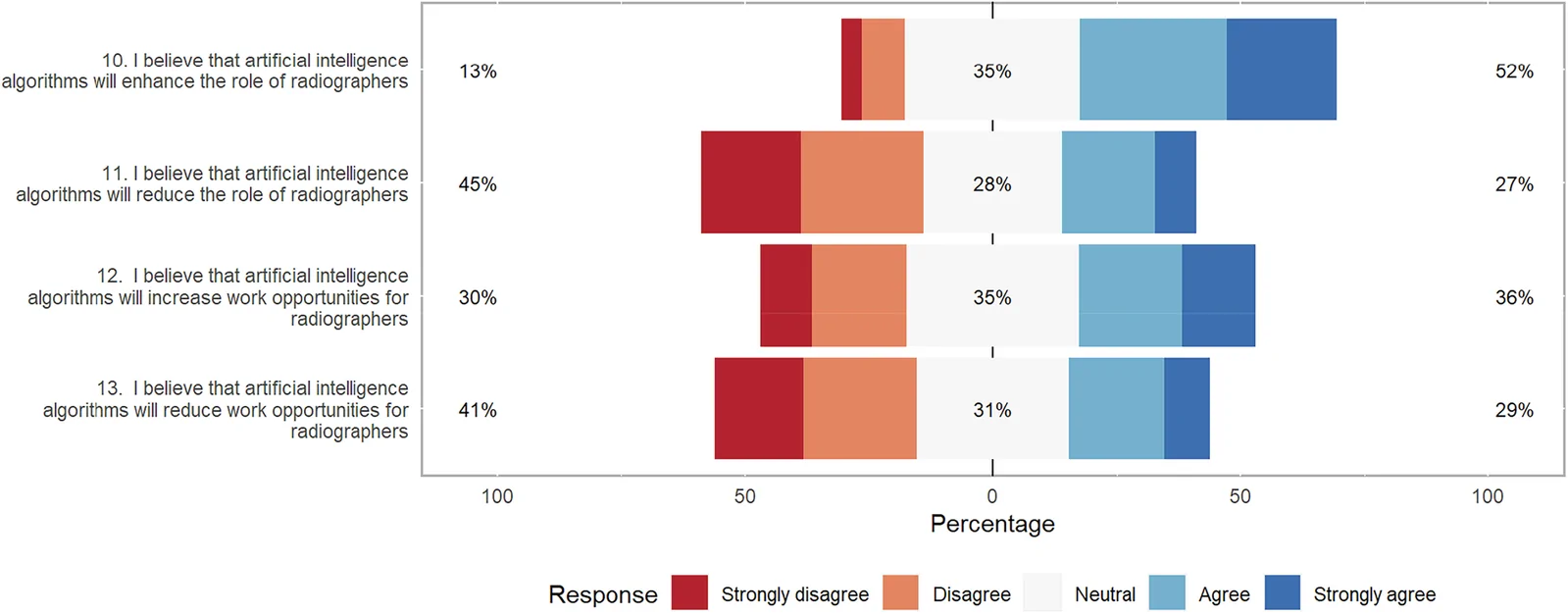
Responses to the questions regarding AI

Views on the impact of AI on the radiographers’ role were heterogeneous. Over half of participants (51.9%, *n* = 804) agreed or completely agreed that AI would strengthen and enhance their professional role, while 35.1% (*n* = 544) remained neutral and 12.9% (*n* = 200) disagreed. Concerns about a potential reduction of the radiographer's role due to AI were less prevalent, with 44.9% (*n* = 695) disagreeing or completely disagreeing with this statement. Expectations regarding employment opportunities were mixed: 35.9% (*n* = 555) anticipated increased opportunities related to AI adoption, whereas 28.7% (*n* = 444) expected a reduction (Fig. 3).

### Impact of the COVID-19 pandemic

**Fig. 4:**
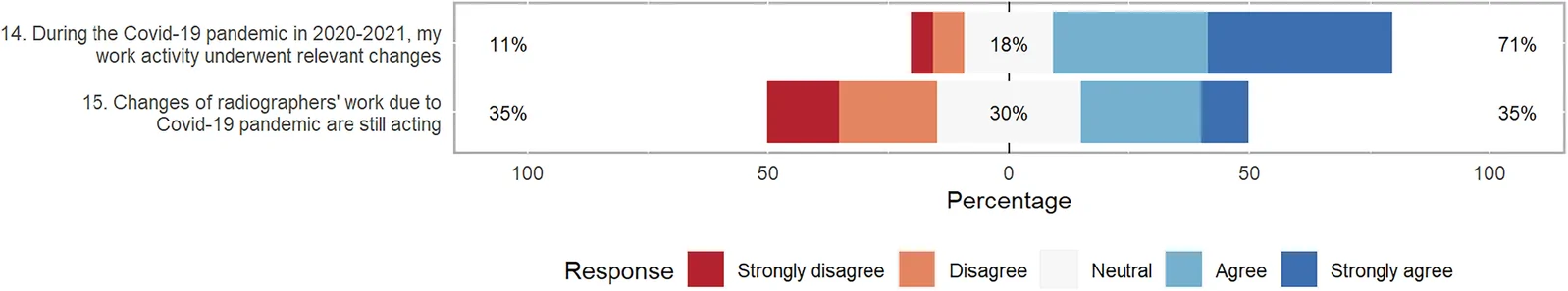
Responses to the questions regarding the COVID-19 pandemic

Most participants (70.6%, *n* = 1093) reported that the COVID-19 pandemic led to significant changes in their professional activity. However, perceptions regarding the persistence of these changes were more evenly distributed: 34.9% (*n* = 540) agreed that changes remained in place, 29.8% (*n* = 461) were neutral, and 35.3% (*n* = 547) disagreed (Fig. 4).

### Education, career development, and professional satisfaction

**Fig. 5:**
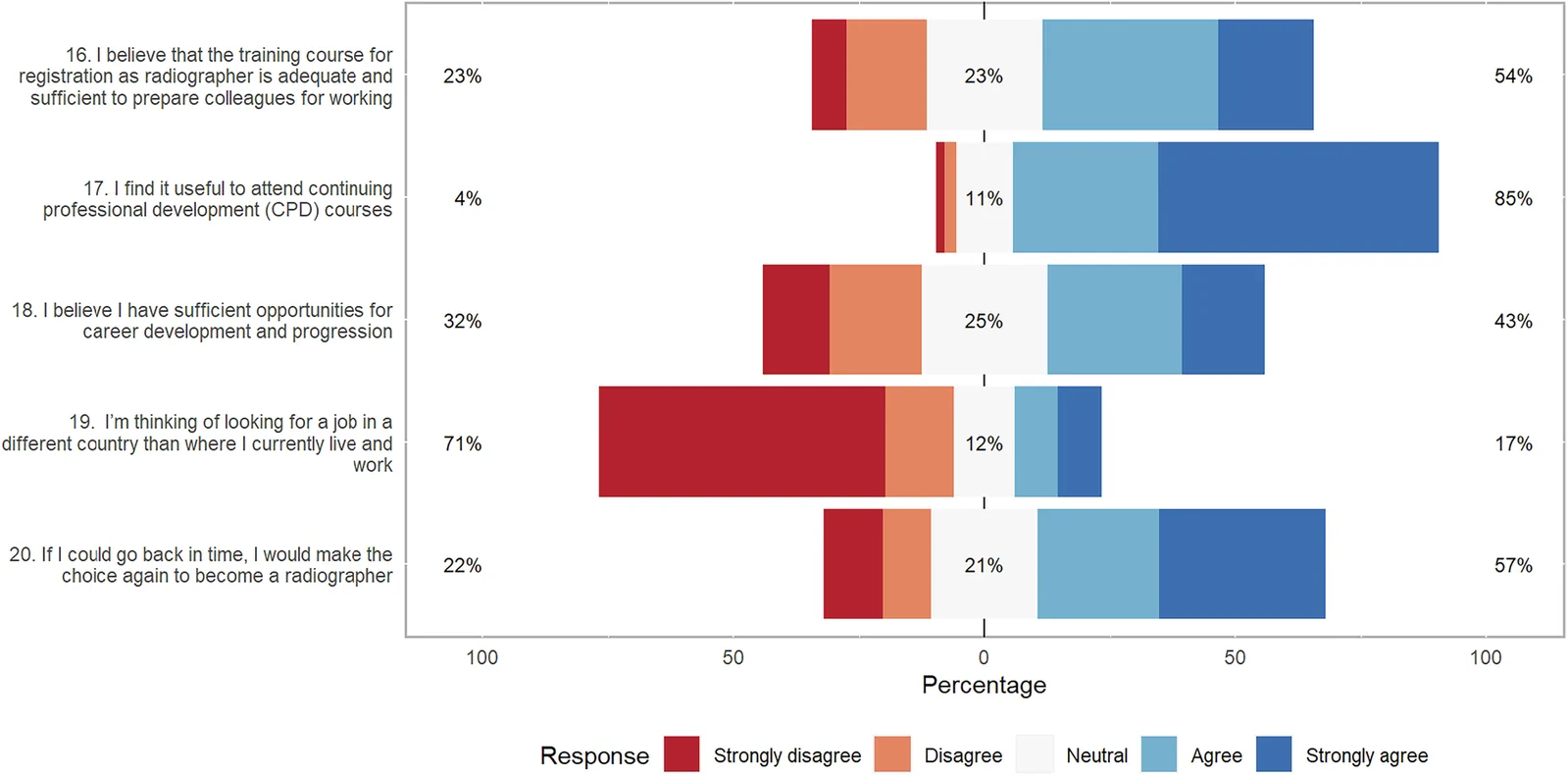
Responses to questions regarding Education and career

Regarding adequacy of initial education, 54.0% (*n* = 836) of participants agreed that their initial training programme for registration as a radiographer provided sufficient preparation for clinical practice, while 23.0% (*n* = 356) were neutral and 23.0% (*n* = 356) disagreed. Continuing professional development was almost universally recognised as essential, with 84.8% (*n* = 1,313) expressing agreement. Perceived opportunities for career progression were as follows: 43.3% (*n* = 671) of participants felt they had sufficient opportunities for advancement, whereas 31.7% (*n* = 490) disagreed; 70.8% (*n* = 1096) reported no intention to seek employment in another country. When asked whether they would choose the profession again, 57.4% (*n* = 888) responded positively, 21.6% (*n* = 334) negatively, and 21.1% (*n* = 326) remained undecided (Fig. 5).

## Discussion

This survey aimed to explore the evolution of radiographers’ roles from a pan-European perspective, including their engagement with research, and their views on AI and future professional development. A total of 1548 radiographers participated from 87 countries and associated islands, representing a broad and diverse cohort worldwide.

### Participant characteristics and professional background

The survey primarily reflects a European professional context, as it was disseminated through ESR and EFRS channels, and 1508/1548 participants (97.4%) were currently working in Europe. A small proportion of participants (40/1548; 2.6%) reported working outside Europe and were retained in the analysis. There was a male-female imbalance in the responses, with 66% of females, as expected because radiography remains a female-dominated profession [21], in contrast with radiology, where only 33.5% of radiologists globally are female [22]. The survey attracted participants from a wide age range, demonstrating that the survey was accessible and important to radiographers at all ages and stages of their careers.

The survey explored the country of birth, study and work as broad metrics for transitions between countries. While the numbers were too small to draw clear conclusions regarding movement for most countries, there was a trend of radiographers born and trained elsewhere moving to work in both the UK and Switzerland, and a net flow of radiographers from the African continent towards Europe. This aligns with the global healthcare workforce crisis, with an anticipated shortage of 18 million health workers by 2030 [23], which is driving increased international migration, particularly from low and middle-income countries to high-income countries with the attraction of higher wages and more career opportunities [24].

Participants worked across multiple clinical imaging and therapeutic domains, but conventional radiography remained the modal activity (49.1%), and CT and MRI were also frequently reported (39.0% and 30.7%, respectively), aligning with the wider evidence on current imaging activity volumes [25].

### Clinical role and perceived autonomy

Importantly, most participants reported a substantial degree of autonomy in their daily clinical practice, with around two-thirds reporting they operate independently from radiologists, radiation oncologists or nuclear medicine physicians. While the majority report autonomy, radiographers form an integral part of patient pathways and care teams, and their areas of practice may impact the degree of autonomy they feel and report, along with their stage of career. Perceived autonomy is likely to vary by field of practice. Radiographers working in conventional radiography and mammography often operate with greater procedural independence, which may contribute to higher autonomy ratings, whereas in highly supervised, team-based environments such as interventional radiology, autonomy is inherently more limited. Future analyses that correlate the modality of practice with perceived autonomy would help clarify these modality-specific differences and support more tailored workforce and education strategies. Matilainen et al explored radiographers’ professional rights in diagnostic radiography and reported that these rights included expertise-based rights to plan, conduct, and assess radiological care, as well as patient advocacy [26]. Most participants stated they are the only healthcare professional in direct contact with the patient during imaging examinations or procedures and are responsible for patient radiation protection, optimising image quality and identifying clinical issues in imaging. This finding may partly reflect the heterogeneity of radiographers’ working environments, as those working in research/academia, radiotherapy, nuclear medicine and interventional radiology are more likely to operate in interdisciplinary settings where patient contact and clinical responsibilities may be shared with other healthcare professionals, such as physicians and nurses. This aligns with a scoping review, which reported that radiographers make clinical decisions before, during and after examinations, but it cited a lack of autonomy, sparse information on clinical requests and a lack of time as factors that negatively impact their clinical decision-making [27]. However, only 41% indicated that they were responsible for organ contouring in radiation therapy, suggesting radiographers feel less autonomous in this area of practice. Changes in radiation therapy to incorporate medical directives into practice may support more advanced practice in this role and create greater autonomy in clinical decision-making [28]. At any rate, this topic, i.e., the autonomy and independence degree in day-by-day radiographers’ work, deserves a more in-depth investigation and analysis in correlation with the imaging modalities practised by the participants, varying in clinical complexity from screening mammography to interventional radiology.

### Research involvement and research capacity

Radiographers work within a rapidly changing profession, driven by technological advances, and are relatively under-represented as researchers [2, 3]. Research is an essential part of being a profession, enabling the creation of a profession-specific evidence base [29]. A quarter of participants reported frequent involvement in clinical research activities, while only one-tenth reported being included as co-authors on publications. These figures should be interpreted with caution, as they likely reflect a selection bias. Individuals who choose to participate in a voluntary professional survey, particularly one disseminated through the ESR and EFRS networks, may be disproportionately more engaged in research, academic activities, and professional development than the broader radiography workforce. In addition, the survey was administered in English, which may have favoured participation by participants with higher English proficiency; in several non-English-speaking countries, this characteristic may correlate with greater academic exposure and research involvement. Finally, both ESR and EFRS communication and dissemination channels are partly mediated through academic institutions and research-active communities, which may further skew the sample toward radiographers working in larger hospitals, universities, or research-oriented settings. This may help explain the relatively high proportion of participants holding a PhD qualification in this sample (4.4%), which should not be interpreted as representative of the wider radiographer workforce. Consequently, the true prevalence of research participation and co-authorship across the overall profession may be lower than observed in this survey. Anyway, increasing the inclusion of evidence and research in curricula has been recommended across Europe [2]. Over half of participants indicated they would like to be more involved in research, which supports the need for radiographers to increasingly contribute to the radiography evidence base [30]. By working collaboratively in multidisciplinary research teams to reflect care pathways, radiographers will be able to ensure that patients benefit from optimised diagnosis and treatment [31].

### AI and technological innovation

AI is being rapidly integrated into radiography practice [32–35], and participants had heterogeneous views on the impact of AI on the radiographer's role. However, AI should be interpreted as part of a broader technological evolution in radiography, including advanced imaging equipment, new acquisition techniques and digital workflows, all of which may influence radiographers’ responsibilities, required competencies and professional development. Just over half of the participants agreed that AI would strengthen and enhance their professional role. There were concerns about a potential reduction of the radiographer's role due to AI, but these were less prevalent. Expectations regarding employment opportunities were mixed: around a third anticipated increased opportunities related to AI adoption, while just under a third predicted a reduction. These results align with previous surveys, which indicate benefits relating to enhanced workflows, but concerns regarding deskilling and over-reliance on AI [6, 36, 37]. Recent evidence from a large international workforce report highlights that employees across sectors are generally ready to adopt AI, while leadership alignment and structured training remain the main barriers to successful implementation [38]. This broader perspective reinforces our findings, suggesting that radiographers demonstrate substantial readiness to engage with AI but require clear governance frameworks and targeted educational support to fully realise its potential in clinical practice [39].

### Impact of the COVID-19 pandemic

Over two-thirds of participants indicated the COVID-19 pandemic led to significant changes in their professional activity. However, perceptions regarding the persistence of these changes were heterogeneous, suggesting that some impacts have been mitigated, while others remain. The COVID-19 pandemic had a profound impact on radiographers [40–44], with diagnostic radiographers serving as frontline members of care teams for those hospitalised with the virus and therapeutic radiographers advancing cancer care for their vulnerable populations [45]. The long-term impacts on healthcare, including waiting lists in services which were halted in the pandemic and increased demands due to long-term sequelae of COVID-19, including long-COVID, remain to this day [5, 46–48].

### Education, career development, and professional satisfaction

Finally, just over half of radiographers reported their degree programme provided sufficient preparation for clinical practice, suggesting a widespread need for upskilling of radiographers post-qualification. Some countries already have preceptorship programmes which bridge this transition in a safe and structured way [10]. However, there is insufficient evidence relating to the most appropriate approach and its effectiveness at present [49]. Harvey-Lloyd et al reported these programmes focussed on the completion of tasks, ignoring the process of transformation into the role of a diagnostic radiographer [50].

### Limitations

There are several limitations of this survey. Firstly, while there was a good response in absolute numbers, this is less than 1% of estimated radiographers globally, and the results may not therefore be entirely generalisable, as highlighted above for research activity and co-authorship. The unequal distribution of responses across countries should also be acknowledged, particularly the large proportion of participants from the UK. Since the UK has more established frameworks for advanced, enhanced and consultant radiographer practice than many other European countries, this may have influenced the overall findings, especially regarding autonomy, role development, research engagement and career progression. Some countries had very small numbers of responses, and to maintain anonymity, the decision was made only to report data on countries with five or more responses. The same rule was applied to responses relating to sex, and data were pooled where necessary for the results. The survey was only distributed online, which provides efficient access, but can be subject to sampling bias, with those who have limited access to digital platforms being excluded. While responses were received from many countries, the numbers within most were too small to support meaningful comparative analysis. The survey was distributed only in English, thereby excluding those who are unable to read or respond in English and potentially favouring participants with higher English proficiency. Dissemination through ESR and EFRS channels may have introduced self-selection bias, favouring participation by radiographers already engaged with professional networks, research, education or technological innovation. The questionnaire did not undergo formal validation or a separate pilot study. However, it was refined through an iterative expert review process involving stakeholders from EuroAIM, EFRS, and ESR to ensure content validity. Despite these limitations, this survey provides valuable insights into the radiography profession and underpins the support and development needed for a future with AI, as well as the need to upskill more radiographers to be involved in research.

## Conclusion

In conclusion, this large *EuroAIM/EFRS* survey highlights a profession in transition, characterised by increasing responsibilities, substantial perceived autonomy, heterogeneous educational support and growing engagement with AI. The findings support the need for structured, internationally comparable career frameworks, targeted AI education, and stronger investment in radiographer-led research capacity. Professional societies, educational institutions and healthcare organisations should work collaboratively to ensure that radiographers are supported not only as technical operators, but as autonomous professionals contributing to patient care, clinical decision-making, research and service innovation in an increasingly complex imaging and radiotherapy environment.

## Supplementary information

ELECTRONIC SUPPLEMENTARY MATERIAL

## Data Availability

The datasets generated and/or analysed during the current study are available from the corresponding author on reasonable request.

## Abbreviations

AI: Artificial Intelligence
EFRS: European Federation of Radiographer Societies
EIBIR: European Institute for Biomedical Imaging Research
ESR: European Society of Radiology
EuroAIM: European Network for the Assessment of Imaging in Medicine

## References

[1] The Society of Radiographers (2022) Available at https://committees.parliament.uk/writtenevidence/43583/pdf/

[2] England A, McNulty JP (2020) Inclusion of evidence and research in European radiography curricula. Radiography 26:S45–S48. 10.1016/j.radi.2020.04.018 32444326

[3] Knight KJ, Beasley M, McConnell J et al (2025) Research culture, barriers and facilitators within the radiography workforce in the UK – results of a national survey. Radiography 31:102959. 10.1016/j.radi.2025.102959 40286676

[4] Harrison G, Martins dos Santos R, Kraus B, Pedersen MRV (2021) Radiographers in ultrasound: Motivation and role expansion. A survey of European Federation of Radiographer Societies (EFRS). Radiography 27:1185–1191. 10.1016/j.radi.2021.07.003 34294506

[5] Alghamdi F, Owen R, Ashton REM et al (2022) Post-acute COVID syndrome (long COVID): What should radiographers know and the potential impact for imaging services. Radiography 28:S93–S99. 10.1016/j.radi.2022.08.009 PMC9468096 36109264

[6] Stogiannos N, Walsh G, Ohene-Botwe B, et al (2025) R-AI-diographers: a European survey on perceived impact of AI on professional identity, careers, and radiographers’ roles. Insights Imaging 16:1–13. 10.1186/S13244-025-01918-6 PMC11832980 39962024

[7] Zanardo M, Visser JJ, Colarieti A et al (2024) Impact of AI on radiology: a EuroAIM/EuSoMII 2024 survey among members of the European Society of Radiology. Insights Imaging 15:240. 10.1186/s13244-024-01801-w PMC11458846 39373853

[8] EFRS (2018) European Qualifications Framework (EQF) Level 6 Benchmarking Document: Radiographers, 2nd edn. EFRS

[9] EFRS (2021) RERP-Radiographer Education, Research, and Practice: 2021–2031. EFRS

[10] McNulty JP, Zarb F, Brady AP et al (2025) Guidelines and recommendations for radiographer education from the EU-REST project. Insights Imaging 16:242. 10.1186/s13244-025-02104-4 PMC12583322 41184567

[11] Zanardo M, Rainford L, McGee A et al (2023) An investigation into academic career pathways across Radiography education centres internationally. Radiography 29:992–999. 10.1016/j.radi.2023.07.010 37634415

[12] Oliveira C, Barbosa B, Couto JG et al (2023) Advanced practice roles amongst therapeutic radiographers/radiation therapists: a European survey. Radiography 29:261–273. 10.1016/j.radi.2022.12.003 36608396

[13] Pedersen MRV, Jensen J, Senior C et al (2023) Reporting radiographers in Europe survey: an overview of the role within the European Federation of Radiographer Society (EFRS) member countries. Radiography 29:1100–1107. 10.1016/j.radi.2023.09.005 37757677

[14] Catania D, Giannotti N, Roletto A, Ryan M-L (2024) Opinions on advanced practice among diagnostic and therapeutic radiographers: Survey results of an European congress of radiology study. Radiography 30:806–812. 10.1016/j.radi.2024.03.001 38513333

[15] Harris R, Paterson A (2016) Exploring the research domain of consultant practice: perceptions and opinions of consultant radiographers. Radiography 22:12–20. 10.1016/j.radi.2015.03.002

[16] Heales CJ, Mills K, Ladd E (2021) Radiographer advanced and consultant practice and community diagnostic hubs–a vision for the future. Radiography 27:S28–S33. 10.1016/j.radi.2021.05.004 34119400

[17] Clarkson M, Snaith B, Hizzett K, Harcus J (2026) Exploring enhanced level practice in radiography: a survey of awareness and implementation across the UK. Radiography 32:103222. 10.1016/j.radi.2025.103222 41177056

[18] Codari M, Melazzini L, Morozov SP et al (2019) Impact of artificial intelligence on radiology: a EuroAIM survey among members of the European Society of Radiology. Insights Imaging 10:105. 10.1186/s13244-019-0798-3 PMC6823335 31673823

[19] Stogiannos N, Malik R, Kumar A et al (2023) Black box no more: a scoping review of AI governance frameworks to guide procurement and adoption of AI in medical imaging and radiotherapy in the UK. Br J Radiol. 10.1259/bjr.20221157 PMC10646619 37747285

[20] https://www.legislation.gov.uk/ (2018) Data Protection Act 2018. https://www.legislation.gov.uk/ukpga/2018/12/contents. Accessed 25 Feb 2026

[21] Nightingale J, Appleyard R, McNamara J et al (2022) Gender diversity in therapeutic radiography: A mixed methods exploration of the gender influences impacting on male students’ career choices. Radiography 28:258–266. 10.1016/j.radi.2022.02.005 35221211

[22] Cater SW, Yoon SC, Lowell DA et al (2018) Bridging the Gap. Acad Radiol 25:1052–1061. 10.1016/j.acra.2017.12.021 29398433

[23] World Health Organization (2026) Global strategy on human resources for health: workforce 2030. https://iris.who.int/server/api/core/bitstreams/3ef6ee65-42fa-4d2b-9c75-d55b2df17f9a/content. Accessed 19 Feb 2026

[24] Omiyi D, Snaith B, Iweka E, Wilkinson E (2024) Mapping the migrant diagnostic radiographers in the UK: A national survey. Radiography 30:1713–1718. 10.1016/j.radi.2024.08.015 39244456

[25] Medical Imaging Statistics and Facts (2026) https://media.market.us/medical-imaging-statistics/. Accessed 19 Feb 2026

[26] Matilainen K, Ahonen S, Kankkunen P, Kangasniemi M (2017) Radiographers’ perceptions of their professional rights in diagnostic radiography: a qualitative interview study. Scand J Caring Sci 31:139–145. 10.1111/scs.12335 27164407

[27] Diaby LF, Debess J, Teli M (2024) Clinical decisions-making within diagnostic radiography–a scoping review. Radiography 30:1136–1143. 10.1016/j.radi.2024.05.008 38820676

[28] Javor J, Lavergne C (2024) Empowering radiation therapists through medical directives: a progressive step in radiation therapy. J Med Imaging Radiat Sci 55:S13–S14. 10.1016/j.jmir.2024.03.037 40383108

[29] Stewart-Lord A, Matthews K, Partner A, Mussmann B (2026) Radiographer research engagement: an international mixed methods study. Radiography 32:103364. 10.1016/j.radi.2026.103364 41825210

[30] Al Balushi H, Watts H, Akudjedu TN (2024) Research and evidence-based practice in clinical radiography: a systematic review of barriers and recommendations for a new direction. Radiography 30:538–559. 10.1016/j.radi.2024.01.012 38290178

[31] Di Michele L, Johnson N, McDermott B et al (2026) Exploring how research skills are taught and assessed in medical imaging and radiation therapy higher education: an international snapshot. Radiography 32:103440. 10.1016/j.radi.2026.103440 42143421

[32] Potočnik J, Thomas E, Kearney D et al (2025) Can ChatGPT and Gemini justify brain CT referrals? A comparative study with human experts and a custom prediction model. Eur Radiol Exp 9:24. 10.1186/s41747-025-00569-y PMC11836243 39966263

[33] Lai Q, Chen W, Ding X et al (2024) Quality control of elbow joint radiography using a YOLOv8-based artificial intelligence technology. Eur Radiol Exp 8:107. 10.1186/s41747-024-00504-7 PMC11415556 39302546

[34] Melazzini L, Bortolotto C, Brizzi L et al (2025) AI for image quality and patient safety in CT and MRI. Eur Radiol Exp 9:28. 10.1186/s41747-025-00562-5 PMC11847764 39987533

[35] Albano D, Monti CB, Blanda G et al (2026) MRI-based scoring system to predict spondylodiscitis: The SPONDY-Score. Eur J Radiol 195:112600. 10.1016/j.ejrad.2025.112600 41380377

[36] Arkoh S, Akudjedu TN, Amedu C et al (2025) Current Radiology workforce perspective on the integration of artificial intelligence in clinical practice: a systematic review. J Med Imaging Radiat Sci 56:101769. 10.1016/j.jmir.2024.101769 39437624

[37] Akudjedu TN, Torre S, Khine R et al (2023) Knowledge, perceptions, and expectations of Artificial intelligence in radiography practice: a global radiography workforce survey. J Med Imaging Radiat Sci 54:104–116. 10.1016/j.jmir.2022.11.016 36535859

[38] Sundland SL, Lundvall L-L, Gustafsson H et al (2026) Radiographers’ role in the age of AI: a qualitative comparative multi-case study. Radiography 32:103395. 10.1016/j.radi.2026.103395 41861627

[39] Mayer H, Yee L, Chui M, Roberts R (2025) Superagency in the workplace: Empowering people to unlock AI’s fullpotential. McKinsey & Company. https://elements.visualcapitalist.com/wp-content/uploads/2025/02/superagency-in-the-workplace-empowering-people-to-unlock-ais-full-potential-v3.pdf. Accessed 12 Oct 2026

[40] Zanardo M, Martini C, Monti CB et al (2020) Management of patients with suspected or confirmed COVID-19, in the radiology department. Radiography 26:264–268. 10.1016/j.radi.2020.04.010 PMC7167552 32340912

[41] McNulty JP, Zanardo M, Buissink C et al (2025) Professional challenges faced by student radiographers during COVID-19. Radiography 31:103000. 10.1016/j.radi.2025.103000 40505516

[42] Rainford LA, Zanardo M, Buissink C et al (2020) The impact of COVID-19 upon student radiographers and clinical training. Radiography. 10.1016/j.radi.2020.10.015 PMC7834574 33223416

[43] Zanardo M, Schiaffino S, Sardanelli F (2020) Bringing radiology to patient’s home using mobile equipment: a weapon to fight COVID-19 pandemic. Clin Imaging 68:99–101. 10.1016/j.clinimag.2020.06.031 PMC7301065 32585417

[44] Zanardo M, Cornacchione P, Marconi E et al (2022) Occupational burnout among radiation therapy technologists in Italy before and during COVID-19 pandemic. J Med Imaging Radiat Sci 53:58–64. 10.1016/j.jmir.2021.12.004 PMC8763333 35115275

[45] Mc Fadden S, Flood T, Shepherd P, Gilleece T (2022) Impact of COVID-19 on service delivery in radiology and radiotherapy. Radiography 28:S16–S26. 10.1016/j.radi.2022.03.009 PMC8960154 35422396

[46] Ruiz C, Llopis D, Roman A et al (2021) Spanish radiographers’ concerns about the COVID-19 pandemic. Radiography 27:414–418. 10.1016/j.radi.2020.10.001 PMC7546189 33087309

[47] Naylor S, Booth S, Harvey-Lloyd J, Strudwick R (2022) Experiences of diagnostic radiographers through the Covid-19 pandemic. Radiography 28:187–192. 10.1016/j.radi.2021.10.016 PMC8552557 34736824

[48] Knapp KM, Venner S, McNulty JP, Rainford LA (2022) The risk of burnout in academic radiographers during the COVID-19 pandemic. Radiography 28:1010–1015. 10.1016/j.radi.2022.06.016 PMC9247227 35926446

[49] Chaka B, Singh N, Gallagher S (2024) What does the literature say about preceptorship and mentorship in radiography: A scoping review of the current research and identified knowledge gaps. Radiography 30:1026–1034. 10.1016/j.radi.2024.04.028 38723443

[50] Harvey-Lloyd J, Morris J (2020) Supporting newly qualified diagnostic radiographers: Are we getting it right? Int J Pract-based Learn Health Soc Care 8:57–67. 10.18552/ijpblhsc.v8i2.673

