# Supplementary material for: The evolution of radiographers—an investigation into radiographers’ role and perspectives: a EuroAIM/EFRS survey
Source: Insights Imaging. 2026 Oct 30;17:271. doi: 10.1186/s13244-026-02442-x (PMC13722655; doi:10.1186/s13244-026-02442-x)
Supplement: Supplementary file 1 [file 13244_2026_2442_MOESM1_ESM.pdf]

The evolution of radiographers–an investigation into Radiographers' role and perspectives: a EuroAIM/EFRS survey

ELECTRONIC SUPPLEMENTARY MATERIAL

Section	Item no.	Question text
**Introduction and consent (mandatory)**	–	Participation in this survey is voluntary and anonymous. By continuing, you confirm that you have read the information provided and consent to participate in this study.
**Demographics**	D1	Gender
	D2	Age range (years)
	D3	Birth country
	D4	Where did you study as radiographer (to register as a radiographer)?
	D5	Current country in which you work as a radiographer
	D6	Qualifications awarded
	D7	What are your current prevalent fields of work? (tick all fields that apply)
**Clinical practice**	Q1	1. In the majority of my work, I am independent from the radiologist, the radiation oncologist, or the nuclear medicine physician
	Q2	2. In the majority of my work, I am frequently the only health professional in contact with the patient during the examination/procedure
	Q3	3. In the majority of my work, I believe that I am the professional most responsible for patient radiation protection
	Q4	4. In the majority of my work, I believe that I am the professional most responsible for image quality optimisation
	Q5	5. In the majority of my work, I believe that I am the professional most responsible for organ contouring for radiation therapy
	Q6	6. I feel able to identify clinical critical issues in requests coming from referring physicians to be discussed with the radiologist/nuclear physician/radiation oncologist
**Research involvement**	Q7	7. I am frequently involved in clinical research activities
	Q8	8. I am frequently involved as co-author in scientific publications
	Q9	9. I would like to be more involved in research activities
**Artificial intelligence**	Q10	10. I believe that artificial intelligence algorithms will enhance the role of radiographers

Section	Item no.	Question text
	Q11	11. I believe that artificial intelligence algorithms will reduce the role of radiographers
	Q12	12. I believe that artificial intelligence algorithms will increase work opportunities for radiographers
	Q13	13. I believe that artificial intelligence algorithms will reduce work opportunities for radiographers
**COVID-19 pandemic**	Q14	14. During the Covid-19 pandemic in 2020-2021, my work activity underwent relevant changes
	Q15	15. Changes of radiographers' work due to Covid-19 pandemic are still acting
**Education and career**	Q16	16. I believe that the training course for registration as radiographer is adequate and sufficient to prepare colleagues for working
	Q17	17. I find it useful to attend continuing professional development (CPD) courses
	Q18	18. I believe I have sufficient opportunities for career development and progression
	Q19	19. I’m thinking of looking for a job in a different country than where I currently live and work
	Q20	20. If I could go back in time, I would make the choice again to become a radiographer
**Open comment**	Q21	21. Please add any further comments you may wish to note in the comment box (if necessary, refer to the sentence numbers)
